# Korean Maize Hybrids Present Significant Diversity in Fatty Acid Composition: An Investigation to Identify PUFA-Rich Hybrids for a Healthy Diet

**DOI:** 10.3389/fnut.2020.578761

**Published:** 2020-11-05

**Authors:** Ramesh Kumar Saini, Kannan R. R. Rengasamy, Eun-Young Ko, Jung-Tae Kim, Young-Soo Keum

**Affiliations:** ^1^Department of Crop Science, Konkuk University, Seoul, South Korea; ^2^Institute of Research and Development, Duy Tan University, Da Nang, Vietnam; ^3^Faculty of Environment and Chemical Engineering, Duy Tan University, Da Nang, Vietnam; ^4^Indigenous Knowledge Systems Centre, Faculty of Natural and Agricultural Sciences, North West University, North West Province, South Africa; ^5^Department of Food Science and Biotechnology of Animal Resources, Konkuk University, Seoul, South Korea; ^6^National Institute of Crop Science, RDA, Suwon, South Korea

**Keywords:** polyunsaturated fatty acids, monounsaturated fatty acids, linoleic acid, corn, healthy diet adherence

## Abstract

Precise information on the content and composition of nutritionally essential metabolites in food crops is a prerequisite for dietary recommendations and nutrient-dense food formulations. In the present study, the fatty acid profile of 21 Korean maize hybrids was analyzed by gas chromatography (GC)–mass spectrometry (MS). In the studied hybrids, linoleic acid (LA; C18:2n6c) was dominant (38.0–58.9%), followed by oleic (OA; C18:1n9c) (23.5–45.3%), palmitic (C16:0) (10.8–17.3%), and stearic acids (C18:0) (1.84–3.86%). Among all the quantified fatty acids, the highest variation was recorded for LA and OA. The highest amount of LA (58.9%), the lowest amount of OA (23.5%), and the highest polyunsaturated fatty acids (PUFAs)/monounsaturated fatty acids (MUFAs) ratio of 2.47 were recorded in the Shingwang hybrid. The highest PUFAs/saturated fatty acids (SFAs) ratio of 4.04 was recorded in the Ahndaok hybrid due to the high content of LA (57.8%) and low amount of SFA. Similarly, the highest PUFAs + MUFAs/SFAs ratio of 6.38 was recorded in the Pyeonggangok hybrid as a result of the high OA (34.6%) and LA (51.4%) contents, along with the lowest amount of SFAs. Considering the high levels of health-beneficial MUFAs and PUFAs and low levels of undesirable SFAs, the maize hybrids Pyeonggangok, Ahndaok, and Shingwang can be used in the preparation of a healthy PUFA-rich diet.

**Graphical Abstract:**
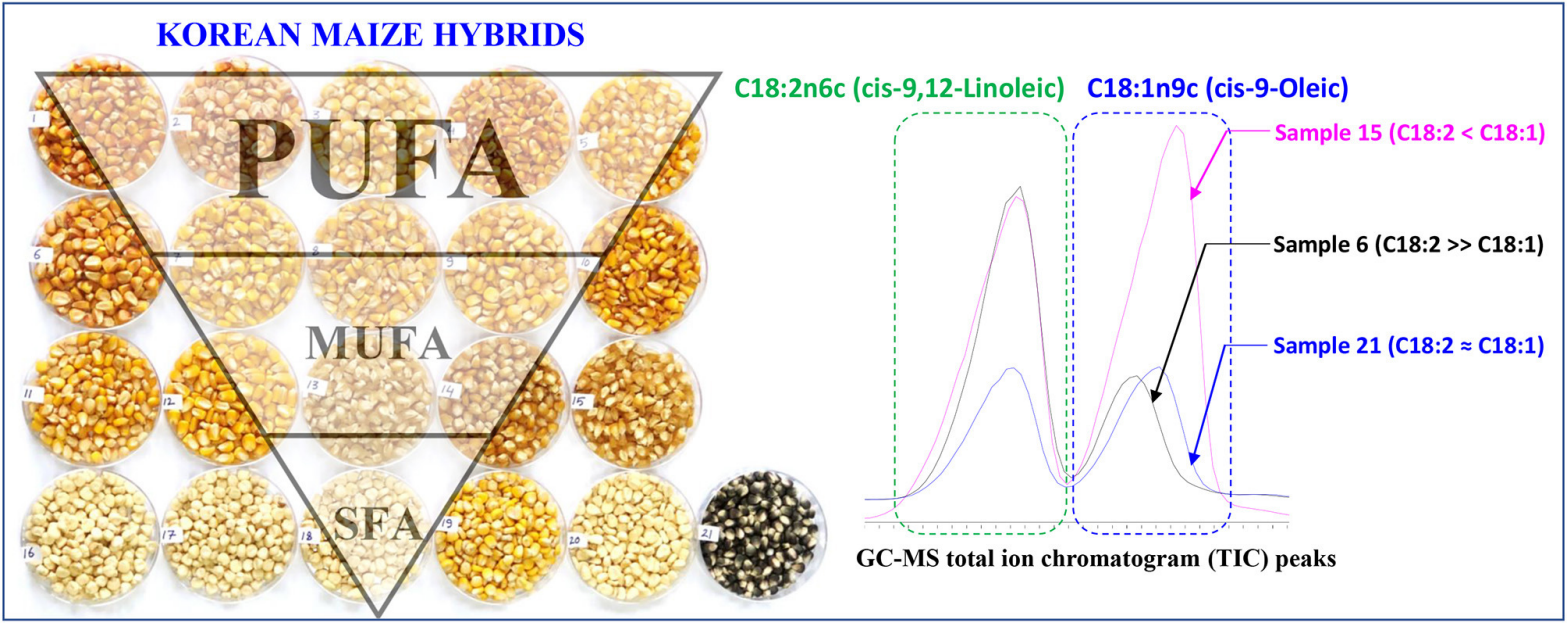


## Introduction

Maize (*Zea mays* L.) is the most demanded grain in the world, with an annual production of over 1 billion metric tons (1). With a global per capita consumption of 135.7 kg/year (202 kg/year in South Korea and 978.4 kg/year in the United States), the maize crop ranked number one in terms of consumption and second in production after sugarcane (1). Starch (52–72%), protein (11–15%), and fats (3–4%) are the significant components of maize grains cultivated for food (2).

Given the critical role of polyunsaturated fatty acids (PUFAs) in positively regulating body homeostasis, a diet rich in PUFAs has gained significant interest in recent years (3). The fatty acid composition of maize grain is more variable in different maize hybrids (4) than that of other significant compounds (e.g., fat, protein, carbohydrates, fiber, amino acids, vitamins, minerals, and secondary metabolites). The fatty acid composition and content in maize is influenced by the genetic background (4–7), fertilizers and irrigation (8), and geographic locations (4, 5, 8). Among these factors, genetic background most significantly influences the composition of nutritionally important primary and secondary metabolites of maize grains, including fatty acids (4). Several quantitative trait loci (QTL) have shown linkages with enzymes in the oil metabolic pathway, responsible for the oil concentration and fatty acid composition (9, 10).

In general, PUFA in the form of linoleic acid (LA; C18:2n6c) represents 35–70% of the total fatty acids found in maize grains, whereas oleic acid (OA; C18:1n9c), a monounsaturated fatty acid (MUFA), represents 23–51% (7, 11–13). Precise assessment of the quantitative and qualitative variability in the fatty acid composition of maize genotypes may yield valuable information concerning health-beneficial fatty acids. Considering these facts, this study aimed to investigate the genetic variation in the fatty acid composition of maize hybrids grown in Korea to identify the hybrids with higher PUFAs contents and low SFAs contents, which may help to precisely recommend the nutritionally dense grains for a healthy diet.

## Materials and Methods

### Plant Material, Reagents, and Standards

**Figure 1:**
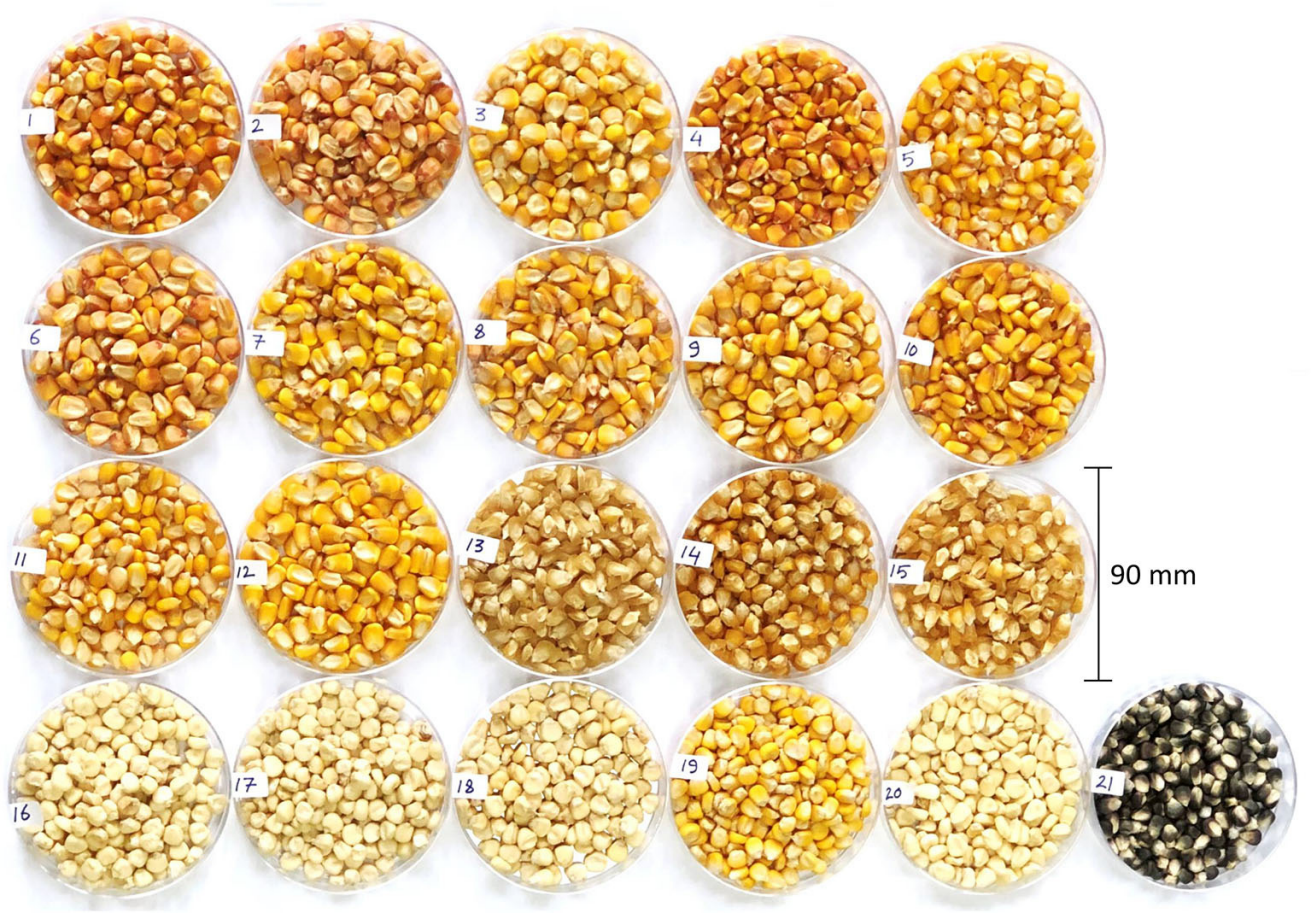
The phenotypic diversity among 21 maize hybrids used in this investigation. 1, Pyeonggangok; 2, Ahndaok; 3, Dachong; 4, Cheongdaok; 5, Pyeongahnok; 6, Shingwang; 7, Shinhwangok; 8, Gwangpyeongok; 9, Hwangdaok; 10, Jangdaok; 11, Gangdaok; 12 Cheongahnok; 13, Suwondan 76; 14, Danok 3; 15, Suwondan 78; 16, Charlok 4; 17, Suwoncharl 81; 18, llmicharl; 19, Hwangmicharl; 20, Mibaek; 21, Heukjinucharl.

The kernels of 21 maize hybrids (Figure 1) used in the present investigation were obtained from the National Institute of Crop Science (NICS), Development Administration (RDA), Suwon, Republic of Korea. The crop was sown in April 2018 and raised following the recommended cultivation practices. The crop was harvested in September 2018 after complete maturity. The kernels were dried naturally, and a 250 g portion of the dried kernels was ground into a powder and stored at −20°C until analysis.

An authentic standard of a fatty acid mix (CRM47885, Supelco 37 Component FAME Mix) was obtained from Merck, Darmstadt, Germany. All organic solvents used for extraction and analysis were of high-pressure liquid chromatography (HPLC) grade and obtained from Daejung Chemicals & Metals Co., Ltd., Korea.

### Extraction and GC–MS Analysis of Fatty Acid Methyl Esters

The crude lipids were extracted from finely powdered maize kernels using chloroform–methanol (2:1 v/v) extraction following our previously optimized method (14), originally based on the findings of Bligh and Dyer (15). Briefly, 1 g of finely powdered maize kernel sample was added to a 50 ml glass tube containing a 25 ml mixture of chloroform/methanol (2:1 v/v) and homogenized using a mechanical homogenizer. The homogenized samples were subjected to bath sonication (300 W, 60 Hz, 40°C) for 10 min for efficient disintegration of the cells and release of the lipophilic components. The extracted samples were centrifuged at 7,000 × *g* for 8 min at 4°C, and the clear supernatant was recovered. The pelleted sample was re-extracted twice with a 15 ml mixture of chloroform and methanol, and the clear supernatants were recovered. The fractions from repeated extractions were pooled in a separating funnel (total volume of 35–40 ml) and partitioned with 0.8 volumes of water containing 1 M sodium chloride (NaCl). The lower chloroform phase containing lipids and other lipophilic compounds (termed crude lipids collectively) was collected into a pre-weighed 250 ml round-bottom flask. The solvent was evaporated using a rotary vacuum evaporator (Büchi, Switzerland) operated at 35°C, and the contents of the crude lipids were determined gravimetrically.

The extracted lipids were converted to fatty acid methyl esters (FAMEs) using methanolic–HCl catalyzed transesterification, according to our optimized method (16). Briefly, 3 ml of methanolic HCl (5% acetyl chloride in methanol, v/v) was added to the flask containing crude lipids, and the contents were transferred to a 20 ml glass vial mounted with a Teflon-reinforced screw cap and incubated in a water bath for 2 h at 55°C. After incubation, the samples were cooled on ice and transferred to a separating funnel, sequentially washed with 5% NaCl and 2% sodium bicarbonate (NaHCO_3_), and the FAMEs were recovered in 10 ml hexane. Subsequently, an aliquot of the sample was syringe-filtered using a 0.45 μm PTFE filter and transferred to a 1.5 ml autosampler vial for gas chromatography (GC)–mass spectrometry (MS) analysis.

The FAMEs were analyzed using a GC–MS-QP2010 SE (Shimadzu, Japan), equipped with a fused silica Rxi-5 ms column (30 m, 0.5 μm film thickness, 0.25 mm ID; Restek Corporation, Bellefonte, PA, USA). The injector and interface were maintained at 260 and 270°C, respectively. Initially, in the first 5 min, the column temperature was maintained at 120°C, then linearly increased to 260°C for 28 min (linear temperature program of 5°C/min), and held at 260°C for 10 min. The column was re-equilibrated at 120°C for 5 min. The FAMEs were precisely identified by comparing their GC–MS retention time and fragmentation pattern with standards (CRM47885).

### Statistical Analysis

All 21 samples were extracted in triplicate and analyzed separately in duplicate, and the values from all six replicates were averaged and presented as means. The data among the 21 studied hybrids were analyzed statistically using SPSS 17.0 software. One-way analysis of variance (ANOVA) was determined with a significance level of *p* < 0.05.

## Results and Discussion

### Korean Maize Hybrids Have a Range of Linoleic and Oleic Acid Levels

**Figure 2:**
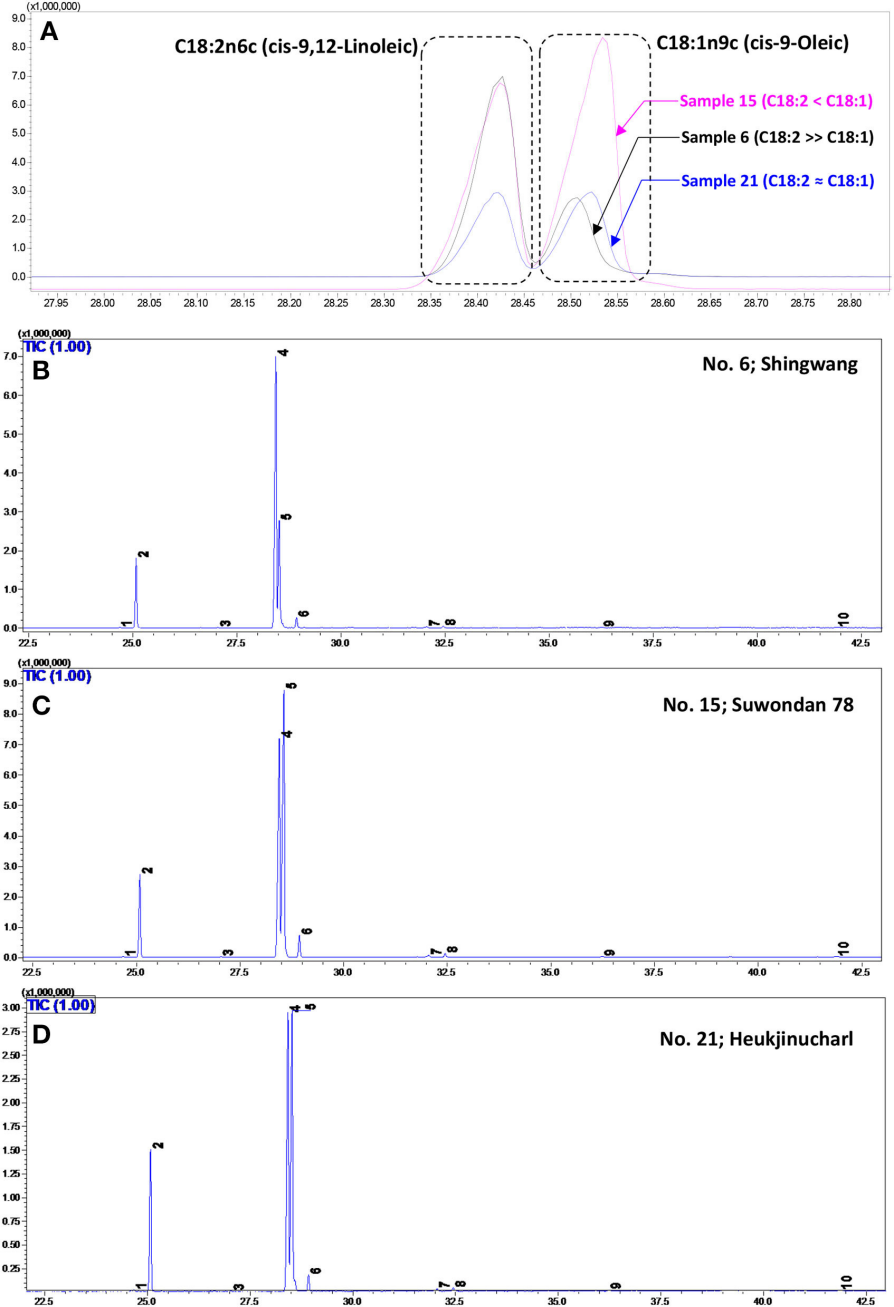
**(A)** A significant variation in the GC–MS total ion chromatogram (TIC) peaks corresponding to linoleic acid (LA; C18:2n6c; peak No. 6) and oleic acid (OA; C18:1n9c; peak No. 4) in the Shingwang (No. 6), Suwondan 78 (No. 15), and Heukjinucharl (No. 21) hybrids is displayed. A representative TIC of major fatty acids identified and quantified from the Shingwang (No. 6) **(B)**, Suwondan 78 (No. 15) **(C)**, and Heukjinucharl (No. 21) hybrids **(D)**.

In the present investigation, 10 fatty acids were identified by GC–MS in the studied 21 maize hybrids (Table 1). The representative GC–MS total ion chromatograms (TICs) with identified peaks are shown in Figure 2. The FAMEs were confirmed by retention times with authentic standards as well as by comparing their fragmentation pattern (Appendix 1, Supplementary Material). In the studied maize hybrids, linoleic acid (LA; C18:2n6c; an n−6 PUFA) was the most abundant (38.0–58.9%), followed by oleic (OA; C18:1n9c) (23.5–45.3%), palmitic (C16:0) (10.8–17.3%), and stearic acids (C18:0) (1.84–3.86%). Palmitoleic (C16:1), heptadecanoic (C17:0), eicosenoic (C20:1n9), arachidic (C20:0), behenic (C22:0), and lignoceric acids (C24:0) accounted for 0.92–2.26% of the total fatty acids. The total crude lipid content was documented on a scale of 3.4 (Dachong; No. 3) to 5.15% (Gangdaok; No. 11).

**Table 1:** Composition of fatty acids in 21 maize hybrids.

**S/no**.	**FAME**	**1**	**2**	**3**	**4**	**5**	**6**	**7**	**8**	**9**	**10**	**11**	**12**	**13**	**14**	**15**	**16**	**17**	**18**	**19**	**20**	**21**
1	C16:1 (cis-9-palmitoleic)	0.11	0.07	0.07	0.10	0.09	0.11	0.12	0.06^b^	0.15	0.15	0.21	0.12	0.14	0.14	0.11	0.15	0.18	0.19	0.24	0.18	0.28^a^
2	C16:0 (palmitic)	10.8^b^	11.8	13.5	13.8	12.9	14.2	13.6	12.7	13.5	12.8	14.6	12.7	11.5	11.4	12.2	14.1	16.0	15.7	16.9	14.8	17.3^a^
3	C17:0 (heptadecanoic)	0.09	0.04	0.05	0.09	0.08	0.17^a^	0.13	0.12	0.10	0.08	0.07	0.07	0.07	0.05	0.06	0.02^b^	0.09	0.04	0.08	0.11	0.05
4	C18:2n6c (LA; cis-9,12-linoleic)	51.4	57.8	49.8	50.6	55.5	58.9^a^	44.8	49.9	49.9	54.0	54.4	50.7	45.0	45.9	38.0	45.6	46.4	48.3	48.0	43.4	38.0^b^
5	C18:1n9c (OA; cis-9-oleic)	34.6	27.7	33.6	31.7	28.6	23.5^b^	37.6	33.6	33.3	29.8	27.6	33.1	39.9	38.0	45.3^a^	35.3	33.8	31.2	31.2	35.6	41.2
6	C18:0 (stearic)	2.10	1.84^b^	1.91	2.66	2.07	2.07	2.80	2.59	2.10	2.32	2.35	2.34	2.35	3.15	3.21	3.24	2.52	3.29	2.37	3.86^a^	2.25
7	C20:1n9 (cis-11-eicosenoic)	0.29	0.12	0.27	0.18	0.21	0.25	0.19	0.21	0.23	0.11^b^	0.16	0.16	0.29	0.34	0.31	0.28	0.24	0.28	0.21	0.44^a^	0.25
8	C20:0 (arachidic)	0.37	0.37	0.45	0.46	0.33	0.42	0.41	0.41	0.39	0.46	0.28^b^	0.47	0.48	0.54	0.54	0.66	0.47	0.55	0.63	0.91^a^	0.46
9	C22:0 (behenic)	0.08^b^	0.16	0.15	0.15	0.10	0.22	0.11	0.15	0.15	0.15	0.15	0.17	0.14	0.16	0.14	0.26	0.17	0.10	0.17	0.31^a^	0.17
10	C24:0 (lignoceric)	0.11	0.12	0.17	0.30	0.11	0.14	0.19	0.21	0.09	0.09	0.13	0.18	0.14	0.27	0.12	0.34	0.19	0.36^a^	0.28	0.34	0.06^b^
	Total SFAs	13.6^b^	14.3	16.3	17.4	15.6	17.2	17.3	16.2	16.3	15.9	17.6	15.9	14.7	15.6	16.2	18.7	19.4	20.1^a^	20.4^a^	20.3^a^	20.3^a^
	Total MUFAs	35.0	27.9	33.9	32.0	28.9	23.8^b^	37.9	33.9	33.7	30.1	28.0	33.3	40.3	38.5	45.7^a^	35.8	34.2	31.6	31.6	36.2	41.7
	Total PUFAs	51.4	57.8	49.8	50.6	55.5	58.9^a^	44.8	49.9	49.9	54.0	54.4	50.7	45.0	45.9	38.0	45.6	46.4	48.3	48.0	43.4	38.0^b^
	PUFAs/SFAs	3.80	4.04^a^	3.07	2.91	3.57	3.45	2.60	3.09	3.06	3.39	3.09	3.19	3.07	2.94	2.36	2.44	2.39	2.41	2.36	2.14	1.87^b^
	PUFAs/MUFAs	1.47	2.07	1.47	1.58	1.92	2.47^a^	1.18	1.47	1.48	1.80	1.95	1.52	1.12	1.19	0.83^b^	1.28	1.36	1.53	1.52	1.20	0.91
	MUFAs/SFAs	2.57	1.95	2.09	1.83	1.85	1.38	2.20	2.09	2.06	1.89	1.59	2.09	2.74	2.47	2.82^a^	1.92	1.77	1.58	1.55^b^	1.78	2.06
	C18:2n6c/C18:1n9c	1.48	2.09	1.48	1.60	1.94	2.51^a^	1.19	1.49	1.50	1.81	1.98	1.54	1.13	1.21	0.84^b^	1.29	1.37	1.55	1.54	1.22	0.92
	PUFAs + MUFAs/SFAs	6.38^a^	5.99	5.16	4.75	5.42	4.84	4.80	5.18	5.12	5.28	4.68	5.28	5.81	5.41	5.19	4.36	4.16	3.99	3.91^b^	3.92	3.93
	Total lipids (% DW)	4.55	4.35	3.40^b^	3.65	4.55	3.45	4.90	3.45	4.60	5.05	5.15^a^	4.35	4.15	4.33	3.78	4.48	3.68	4.55	4.35	4.29	4.34

*Values are percentages of the total fatty acids, from an average of three determinations. Maize hybrids (No. 1–21) correspond with Figure 1. Means accompanied by alphabets “a” and “b” represent the significantly highest and lowest values, respectively, at p < 0.05. SFAs: total saturated fatty acids (C16:0 + C17:0 + C18:0 + C20:0 + C22:0 + C24:0); MUFAs: total monounsaturated fatty acids (C16:1 + C18:1 + C20:1); PUFAs: total polyunsaturated fatty acids (C18:2)*.

Among the studied maize hybrids, the highest variability was recorded for the LA and OA contents. The LA/OA ratio was found in the range of 0.84 (Suwondan 78; No. 15) to 2.51 (Shingwang; No. 6). The LA/OA ratio of 0.84 in the Suwondan 78 hybrid showed high OA (45.3%) content compared with LA (38.0%). Conversely, the highest amount of LA (58.9%) and the lowest amount of OA (23.5%) in the Shingwang hybrid made it the most abundant source of PUFAs, with low levels of MUFAs (highest PUFAs/MUFAs ratio of 2.47). The highest PUFAs/SFAs ratio of 4.04 was recorded in the Ahndaok hybrid (No. 2), attributable to the high content of LA (57.8%) and low amount of saturated fatty acids (SFAs) (e.g., C16:0 and C18:0). Similarly, the highest PUFAs + MUFAs/SFAs ratio of 6.38 was recorded in the Pyeonggangok hybrid (No. 1), owing to the high OA (34.6%) and LA (51.4%) contents with the lowest amount of saturated fatty acids, especially C16:0.

Consistent with the present study, a significant variation in fatty acid composition has been reported previously among the maize hybrids and inbred lines (5–7, 11). Among 418 maize hybrids grown in Iowa, USA, palmitic acid (6.7–16.5%), palmitoleic acid (0.0–1.2%), stearic acid (0.7–6.6%), OA (16.2–43.8%), LA (39.5–69.5%), linolenic acid (0.0–3.1%), and arachidic acid (0.0–1.0%) showed significant variability in composition (5). LA dominated in isogenic and genetically modified (GM) maize kernels cultivated in Spain (45–50% LA) (6). In these isogenic and GM maize cultivars, the LA/OA ratio was found in the range of 1.5–1.8%. Similarly, LA (49.7–62.7%), OA (23.5–34.9%), and palmitic acid (9.5–11.5%) were the predominant fatty acids in three maize varieties cultivated in the USA (11). In the yellow and white kernels from the F2 double haploids of Mexican maize genotypes, OA represented 36–51% of the total fatty acids, and LA ranged from 35 to 52% (7).

### Purple/Black Maize Does Not Represent a Healthy Fatty Acid Profile

High amounts of anthocyanins, especially the glucosides and malonyl-glucosides of cyanidin, pelargonidin, and peonidin provide the black/purple coloration of maize seeds (17). Moreover, the potent antioxidant activities of these polyphenolic compounds significantly enhance the nutritional quality of a diet (17). However, in the present study, among all the studied hybrids, the lowest LA content (38.0%) and the highest contents of low-density lipoprotein (LDL) cholesterol-raising (18) palmitic acid (17.3%) were recorded in the purple corn hybrids (No. 21), with the lowest PUFAs/SFAs ratio of 1.87. Thus, concerning the fatty acid composition, purple maize hybrids do not represent healthy fatty acid profiles.

### Principal Component Analysis

**Figure 3:**
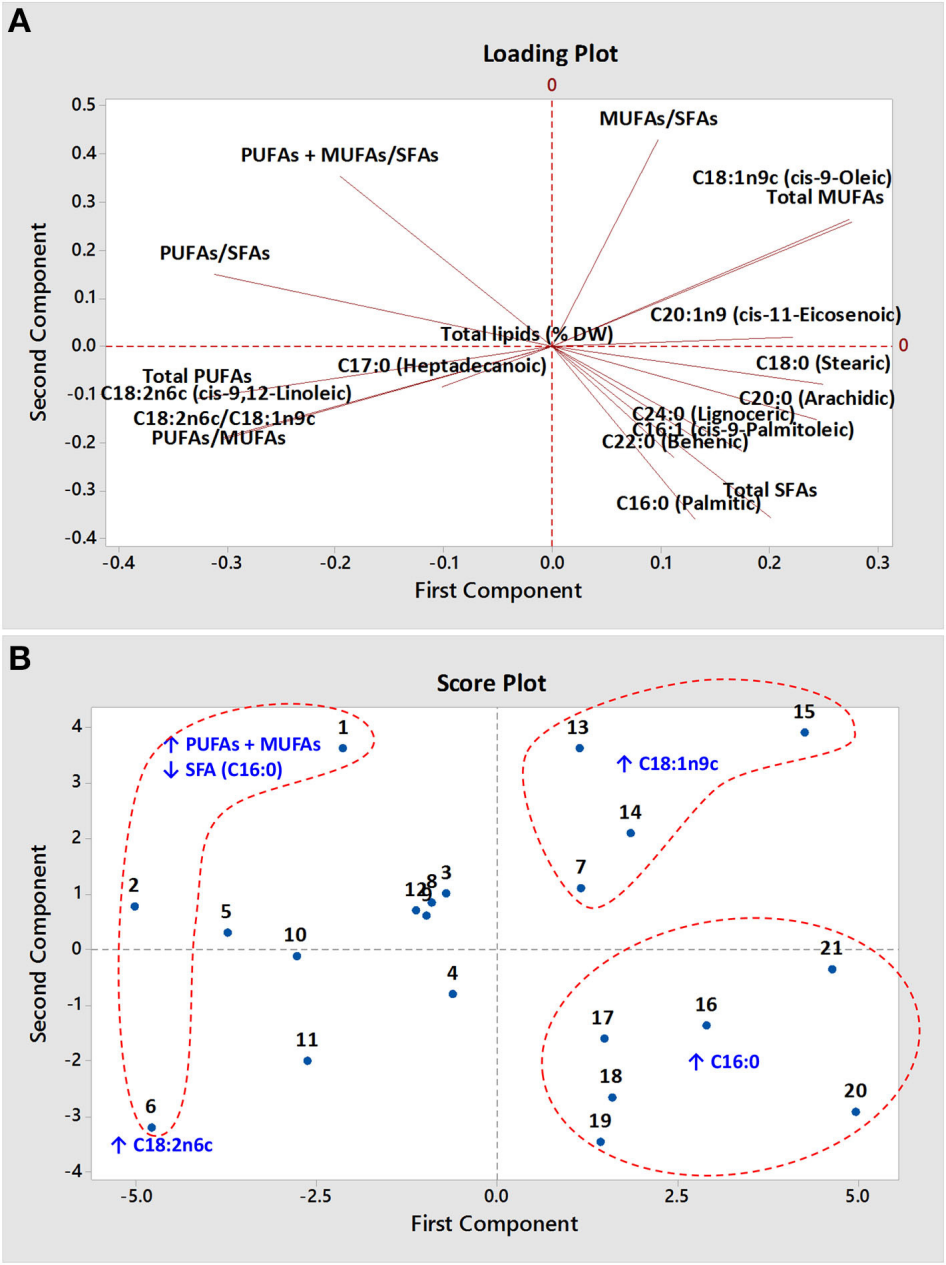
The loading plot **(A)** and score plot **(B)** of 21 maize hybrids. The upwards direction (↑) and downwards direction (↓) arrows represent the higher contents and lower contents, respectively.

The principal component analysis (PCA) of the fatty acid composition data yielded four principal components (PCs) with eigenvalues ≥1 that accounted for 88.3% of the total variance (Appendix 2, Supplementary Material). The first two PCs, PC-1 and PC-2, contributed 45 and 25.6% of the total recorded variance, respectively. Eigen analysis of the covariance matrix loadings of the first two PCs revealed that LA mainly contributed to PC-1, whereas OA and C16:0 mainly contributed to PC-2 (Appendix 2, Supplementary Material). The loading plot (Figure 3A) and the correlation matrix (Appendix 3, Supplementary Material) showed strong negative correlations (*r*^2^ = −0.93) between OA and LA, whereas palmitic and stearic acids correlated positively with palmitoleic and arachidic acids, respectively. Ortíz-Islas et al. (7) also observed a high negative correlation (−0.890 and −0.890 in yellow and white high-oil kernels, respectively) between OA and LA contents.

The score plot generated using fatty acid composition data of studied maize hybrids showed prominent grouping according to their fatty acids profile (Figure 3B). The cluster of maize hybrids Nos. 7, 13, 14, and 15 in the upper right-hand quadrant of the score plot may be explained by their richness of MUFAs (OA). Similarly, the hybrids (Nos. 1, 2, 6) with the higher levels of PUFAs (LA) and the low levels of SFAs (palmitic) were found to be distinctly separated from the other studied hybrids. The cluster of maize hybrids with higher levels of palmitic acid was also distinctly grouped in the lower right-hand quadrant of the score plot.

### Maize Hybrids With Desired Fatty Acid Compositions

Several carefully conducted human intervention trials have shown that compared with MUFAs or PUFAs, diets rich in SFA significantly increased total and LDL cholesterol levels (18). However, LDL cholesterol-raising potency varies significantly with the carbon chain length of SFAs. For instance, lauric (C12), myristic (C14), and palmitic acids (C16) are the most potent in augmenting LDL cholesterol, type 2 diabetes, and cardiovascular disease (CVD), whereas stearic acid (C18) is associated with the lowest or no effects on LDL cholesterol and CVD incidence (18, 19). Lauric and myristic acids are predominantly found in edible coconut and palmolein oil, whereas they are rarely found in edible oils obtained from maize, cotton seeds, mustard, rice bran, safflower, sunflower, and soybean (20). However, these edible oils contain a significant amount of palmitic acids, with the highest in cotton seeds (23.40%) and rice bran oil (19.34%) and a moderate level (12.94%) in maize oil (20). In the present study, the lowest levels (10.8%) of palmitic acid were recorded from hybrid Pyeonggangok (No. 1).

The dietary guidelines recommend <10% of calories (energy) from SFAs, 2.5–10% from n−6 PUFAs (e.g., LA), and 0.5–2% from n−3 PUFAs (21). Moreover, nutritionists are endorsing the replacement of SFAs with PUFAs and MUFAs (22). Replacing the SFAs (especially lauric, myristic, and palmitic acids) with MUFAs (e.g., OA) and PUFAs (e.g., LA) demonstrated LDL cholesterol-lowering effects, along with other many health benefits (18). Thus, the incorporation of appropriate levels of MUFAs and PUFAs with enhanced levels of long-chain (LC)-PUFAs (e.g., eicosapentaenoic acid and docosahexaenoic acid) is recommended for improved health benefits (18). No precise recommendations are existing for the ideal ratio of dietary PUFAs and MUFAs over SFAs. However, based on the recommended unsaturated fatty acids (UFAs: PUFAs + MUFAs) and SFAs daily intakes, MUFAs/SFAs ratio of ~1, PUFAs/MUFAs ratio of ~2, and PUFAs + MUFAs/SFAs ratio of 1.6–2 are considered good (13, 23). Based on the fatty acid profile (Table 1), it can be concluded that Korean maize hybrid Ahndaok (No. 2) and several other hybrids are having an ideal PUFAs/MUFAs ratio. On the other hand, due to the low levels of SFAs in all the studied hybrids, MUFAs/SFAs and PUFAs + MUFAs/SFAs ratio is found to be higher than these recommendations. Nevertheless, it cannot be treated unfavorably, as recent reports are suggesting a reduction in SFAs consumption as much as possible (22).

The results of the present investigation suggest that oil from Korean maize hybrids possesses good qualities in terms of significantly low levels of SFAs and higher levels of MUFAs and PUFAs (24). Considering the highest levels of PUFAs (51.4–58.9%) and the low levels of SFAs (13.6–17.2%), maize hybrids Pyeonggangok (No. 1), Ahndaok (No. 2), and Shingwang (No. 6) can be used in the preparation of a PUFA-rich diet. However, these recommendations are based only on fatty acids content, as other hybrids might be richer in other health-beneficial dietary components.

Given the strong negative correlation (*r*^2^ = −0.93) observed between the OA and LA contents, the selection of hybrids with higher levels of both OA and LA is challenging. Maize oil is preferred mainly for deep frying, due to its high oxidative stability, high smoke point, minimum color darkening during use, and distinctive flavor (25). Because of the nutritional perspective associated with oxidative stability, oils with low levels of SFAs and PUFAs and higher levels of MUFAs are preferred (25). Among the maize hybrids studied in the present investigation, oil from Suwondan 78 (No. 15) may represent the highest oxidative stability, due to the significantly highest levels of OA (45.3%), with low LA (38.0%) levels. Moreover, the oil from maize kernels is considered a rich source of antioxidant compounds, including tocopherols (mainly γ- and α-tocopherol) and phytosterols (24, 26). Higher levels of these antioxidant compounds in maize kernels impart improved health benefits. Additionally, the presence of a substantial amount of these antioxidants in maize oil may improve storage stability and thermal stability (24).

## Conclusions

Due to the high MUFAs and PUFAs levels and low SFAs levels, oil extracted from the maize hybrids Pyeonggangok (No. 1), Ahndaok (No. 2), and Shingwang (No. 6) can be promoted for the preparation of a healthy PUFA-rich diet, whereas the significantly highest levels of OA (45.3%), with a low level of LA (38.0%) in the Suwondan 78 hybrid (No. 15), which may represent high oxidative stability, can be preferred for deep frying. The results of the present investigation suggest that significant variation exists in the fatty acid composition of Korean maize hybrids, which can be utilized to produce oils with desirable fatty acid compositions. In the future, the comprehensive investigations of the composition of other key bioactives from these hybrids can substantially help to make more precise recommendations for a healthy diet.

## Data Availability Statement

The original contributions presented in the study are included in the article/Supplementary Material, further inquiries can be directed to the corresponding author/s.

## Author Contributions

RS: designing, analysis and writing. KR: writing, editing and responsible for submission. E-YK and J-TK: editing the final version. Y-SK: editing and supervision. All authors contributed to the article and approved the submitted version.

## Conflict of Interest

The authors declare that the research was conducted in the absence of any commercial or financial relationships that could be construed as a potential conflict of interest.
