# Supplementary material for: Korean Maize Hybrids Present Significant Diversity in Fatty Acid Composition: An Investigation to Identify PUFA-Rich Hybrids for a Healthy Diet
Source: Front Nutr. 2020 Nov 5;7:578761. doi: 10.3389/fnut.2020.578761 (PMC7678481; doi:10.3389/fnut.2020.578761)
Supplement: Supplementary file 1 [file Data_Sheet_1.DOCX]

**Supplementary file**

Appendix 1. The GC-Mass spectrum of fatty acid methyl esters identified in maize cultivars

**Appendix 1** (cont.) The GC-Mass spectrum of fatty acid methyl esters identified in maize cultivars

**Appendix 2**. Loadings for the first four principal components (PC) of the fatty acids quantified from 21 maize hybrids.

| **S/No.** | **Variable** | **PC1** | **PC2** | **PC3** | **PC4** |
| --- | --- | --- | --- | --- | --- |
| 1 | C16:1 (cis-9-Palmitoleic) | 0.175 | -0.217 | 0.397 | 0.109 |
| 2 | C16:0 (Palmitic) | 0.131 | -0.357 | 0.277 | -0.201 |
| 3 | C17:0 (Heptadecanoic) | -0.102 | -0.083 | -0.159 | -0.532 |
| 4 | C18:2n6c (cis-9,12-Linoleic) | -0.327 | -0.106 | -0.09 | 0.094 |
| 5 | C18:1n9c (cis-9-Oleic) | 0.274 | 0.264 | 0.054 | -0.069 |
| 6 | C18:0 (Stearic) | 0.25 | -0.078 | -0.287 | 0.276 |
| 7 | C20:1n9 (cis-11-Eicosenoic) | 0.222 | 0.021 | -0.352 | -0.048 |
| 8 | C20:0 (Arachidic) | 0.244 | -0.151 | -0.329 | 0.182 |
| 9 | C22:0 (Behenic) | 0.112 | -0.23 | -0.341 | 0.003 |
| 10 | C24:0 (Lignoceric) | 0.148 | -0.21 | -0.353 | 0.294 |
| 11 | Total SFAs | 0.201 | -0.354 | 0.114 | -0.085 |
| 12 | Total MUFAs | 0.277 | 0.259 | 0.052 | -0.068 |
| 13 | Total PUFAs | -0.327 | -0.106 | -0.09 | 0.094 |
| 14 | PUFAs/SFAs | -0.311 | 0.15 | -0.111 | 0.123 |
| 15 | PUFAs/MUFAs | -0.304 | -0.19 | -0.07 | 0.01 |
| 16 | MUFAs/SFAs | 0.098 | 0.431 | -0.039 | 0.014 |
| 17 | C18:2n6c/C18:1n9c | -0.303 | -0.192 | -0.071 | 0.009 |
| 18 | PUFAs + MUFAs/SFA | -0.196 | 0.355 | -0.111 | 0.104 |
| 19 | Total lipids (% DW) | -0.023 | -0.018 | 0.338 | 0.637 |
|  | Eigen Value | 8.5441 | 4.8674 | 2.1109 | 1.2526 |
|  | Variance | 0.45 | 0.256 | 0.111 | 0.066 |
|  | Cumulative | 0.45 | 0.706 | 0.817 | 0.883 |

**Appendix 3.** Correlation matrix of major fatty acids quantified in studied maize cultivars.

| Fatty acid | C16:1 | C16:0 | C17:0 | C18:2n6c | C18:1n9c | C18:0 | C20:1n9 | C20:0 | C22:0 | C24:0 |
| --- | --- | --- | --- | --- | --- | --- | --- | --- | --- | --- |
| C16:1 | 1.00 |  |  |  |  |  |  |  |  |  |
| C16:0 | 0.76 | 1.00 |  |  |  |  |  |  |  |  |
| C17:0 | -0.21 | 0.00 | 1.00 |  |  |  |  |  |  |  |
| C18:2n6c | -0.43 | -0.26 | 0.29 | 1.00 |  |  |  |  |  |  |
| C18:1n9c | 0.16 | -0.11 | -0.31 | -0.93 | 1.00 |  |  |  |  |  |
| C18:0 | 0.22 | 0.15 | -0.12 | -0.58 | 0.44 | 1.00 |  |  |  |  |
| C20:1n9 | 0.13 | 0.03 | -0.02 | -0.55 | 0.49 | 0.63 | 1.00 |  |  |  |
| C20:0 | 0.31 | 0.30 | -0.15 | -0.52 | 0.33 | 0.77 | 0.67 | 1.00 |  |  |
| C22:0 | 0.20 | 0.30 | 0.06 | -0.14 | -0.05 | 0.43 | 0.40 | 0.71 | 1.00 |  |
| C24:0 | 0.11 | 0.27 | -0.15 | -0.20 | 0.00 | 0.74 | 0.42 | 0.70 | 0.42 | 1.00 |
